# Molecular signatures of neural connectivity in the olfactory cortex

**DOI:** 10.1038/ncomms12238

**Published:** 2016-07-18

**Authors:** Assunta Diodato, Marion Ruinart de Brimont, Yeong Shin Yim, Nicolas Derian, Sandrine Perrin, Juliette Pouch, David Klatzmann, Sonia Garel, Gloria B Choi, Alexander Fleischmann

**Affiliations:** 1 Center for Interdisciplinary Research in Biology (CIRB), Collège de France, and CNRS, UMR 7241 and INSERM U1050, F-75005 Paris, France; 2 Department of Brain and Cognitive Sciences, McGovern Institute for Brain Research, Massachusetts Institute of Technology, Cambridge, Massachusetts 02139, USA; 3 Sorbonne Universités, UPMC Univ Paris 06, INSERM U959, Immunology-Immunopathology-Immunotherapy (I3), and AP-HP, Clinical Investigation Center in Biotherapy, Hôpital Pitié-Salpêtrière, F-75013 Paris, France; 4 École Normale Supérieure, Institut de Biologie de l'ENS, Plateforme Génomique, and INSERM U1024, CNRS UMR 8197, F-75005 Paris, France; 5 École Normale Supérieure, Institut de Biologie de l'ENS, and INSERM U1024, CNRS UMR 8197, F-75005 Paris, France

## Abstract

The ability to target subclasses of neurons with defined connectivity is crucial for uncovering neural circuit functions. The olfactory (piriform) cortex is thought to generate odour percepts and memories, and odour information encoded in piriform is routed to target brain areas involved in multimodal sensory integration, cognition and motor control. However, it remains unknown if piriform outputs are spatially organized, and if distinct output channels are delineated by different gene expression patterns. Here we identify genes selectively expressed in different layers of the piriform cortex. Neural tracing experiments reveal that these layer-specific piriform genes mark different subclasses of neurons, which project to distinct target areas. Interestingly, these molecular signatures of connectivity are maintained in *reeler* mutant mice, in which neural positioning is scrambled. These results reveal that a predictive link between a neuron's molecular identity and connectivity in this cortical circuit is determined independent of its spatial position.

## Abstract (web summary)

The piriform cortex projects to multiple brain regions involved in diverse aspects of olfactory behavior but information about the organization of these outputs is lacking. Here the authors show that piriform neurons exhibit layer specific gene expression patterns that also define distinct projection targets.

## Graphical abstract

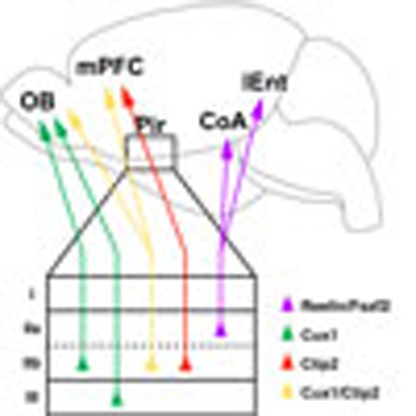

Sensory stimuli in the environment are detected by neurons in the periphery, and stimulus-evoked neural activity is then transmitted to higher brain structures, which extract feature information and reconstruct a coherent sensory percept. Olfactory stimuli are detected by odorant receptors, expressed on the dendrites of olfactory sensory neurons in the nose. In the mouse, each sensory neuron expresses one of ∼1,000 odorant receptor genes^1^,^2^,^3^, and neurons expressing a given receptor project with precision to one of the two spatially stereotyped glomeruli in the olfactory bulb (OB)^4^,^5^,^6^. Mitral and tufted cells, the main projection neurons of the OB, extend a single dendrite into only one glomerulus, and project axons to a number of higher brain regions, including the piriform cortex, the anterior olfactory nucleus (AON), the olfactory tubercle (OT), the cortical amygdala and the lateral entorhinal cortex (lENT)^7^. Projections from individual glomeruli to the piriform cortex are widespread and diffuse^8^,^9^,^10^, and single piriform neurons receive convergent inputs from multiple glomeruli^11^,^12^, suggesting that stimulus features may be reconstructed in the piriform cortex to form odour percepts.

Odour information encoded by piriform ensembles must then be transmitted to brain areas involved in multimodal integration, cognition and motor control. Several target areas of the piriform cortex have been described, including the AON, the OT, the cortical amygdala and the lENT, which themselves receive direct input from the OB^7^,^13^. Piriform neurons also project to the mediodorsal nucleus of the thalamus, to several subdivisions of the prefrontal cortex (PFC) including the infralimbic, orbitofrontal and agranular insular cortex and form strong feedback connections with the OB^7^,^14^,^15^,^16^,^17^. However, how this efferent connectivity is organized at the cellular level remains unknown.

Insights into the specificity of neural connections can be gleaned from gene expression patterns. In the spinal cord, for example, a molecular code of transcription factors and the expression of cell surface proteins distinguish different motor neuron subtypes. Such molecularly defined motor neuron subtypes are clustered into spatially segregated motor pools and project with precision to their cognate muscle targets^18^,^19^. In the neocortex, recent experiments have highlighted the significance of layer-specific genes in delineating the molecular identity of different subclasses of cortical projection neurons^20^. For example, Cux2 and Satb2 expression in neurons localized in the superficial neocortical layers II/III mark callosal projection neurons^21^,^22^,^23^, while subcerebral projection neurons in layer Vb of the motor cortex selectively express Fezf2 (refs 24, 25, 26).

To reveal the molecular identity and connectivity of subclasses of piriform neurons, we decided to identify layer-specific genes in the piriform cortex. The piriform cortex is an evolutionarily ancient paleocortical structure, consisting of only three layers, compared with the six layers typically observed in the neocortex. Layer I contains the axon terminals of OB mitral and tufted cells, the apical dendrites of layer II/III pyramidal and semilunar cells, and a sparse population of local interneurons^27^,^28^. Layer II comprises densely packed excitatory pyramidal and semilunar cells, while layer III harbours a more heterogeneous population of excitatory and inhibitory neurons^29^. We used laser-capture micro-dissection and RNA deep sequencing to identify genes, whose expression is confined to the individual layers of the piriform cortex. Furthermore, we carried out anterograde and retrograde neural tracing experiments to characterize the anatomical organization of distinct subclasses of piriform projection neurons. Combining results from gene expression and neural tracing experiments revealed that layer-specific genes, individually or in combination, could discriminate between different subclasses of projection neurons, independent of whether they were segregated into distinct piriform layers or intermingled within a layer. Finally, we show that these molecular signatures of connectivity are maintained in the absence of piriform layer cytoarchitecture, a scenario observed in Reelin-deficient mice. Our results begin to reveal a molecular code of connectivity in the olfactory cortex and provide important new means of access into the functional and genetic dissection of olfactory circuits.

## Results

### Identification of layer-specific piriform genes

**Figure 1:**
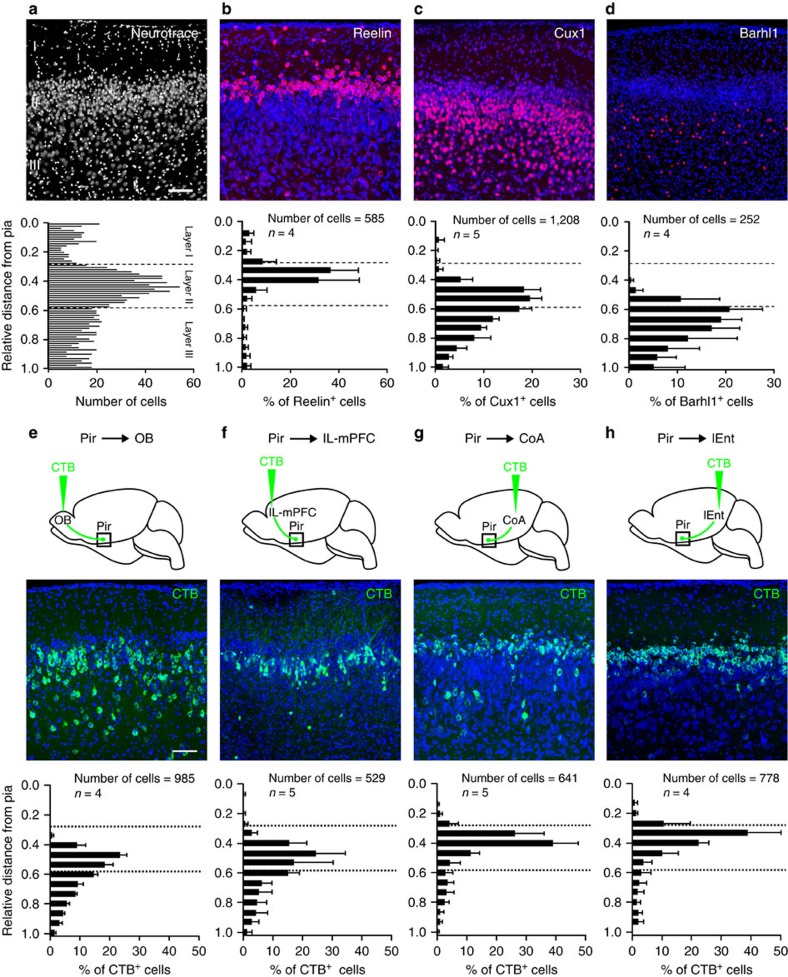
Piriform genes and connections segregate into distinct cortical layers. (**a**) Piriform layers: neurons are sparse in layer I, dense in layer II, and of intermediate density in layer III. (**b**–**d**) Immunohistochemical detection (in red) of proteins differentially expressed across piriform layers. (**b**) Reelin marks densely packed neurons in layer IIa. (**c**) Cux1 is expressed in neurons of layers IIb and III. (**d**) Barhl1 marks subpopulations of neurons in layer III. Neurotrace counterstain in blue. (**e**–**h**) Schematic representations of the cholera toxin B (CTB) injection sites, and coronal sections through the piriform cortex. CTB labelling in green, Neurotrace counterstain in blue. (**e**) Neurons projecting to the olfactory bulb (OB) are present in large numbers in layers IIb and III, while (**f**) neurons projecting to infralimbic division of the medial prefrontal cortex (IL-mPFC) are predominantly located in layer IIb. (**g**,**h**) Neurons projecting to the posteriormedial cortical amygdaloid nucleus (CoA) and the lateral entorhinal cortex (lENT) are enriched in layer IIa. Scale bars, 100 μm. Differential distributions of cells are quantified in histograms, data are represented as mean ±s.d. *n*=number of mice.

We hypothesized that layer-specific piriform genes could reveal a molecular code of projection neuron connectivity. We thus used laser-capture micro-dissection and RNA deep sequencing to identify genes selectively expressed in piriform layers I, II and III. Piriform layers can easily be identified morphologically, based on the differential density of neurons within each layer (Fig. 1a). We then prepared RNA from the micro-dissected tissue and performed RNA deep sequencing to identify genes selectively expressed in neurons of a given layer. DESeq analysis and Independent Component Analysis combined with Gene Set Enrichment Analysis^30^ were used to generate molecular signatures that discriminate the three layers, and to rank genes based on their relative expression levels (see Methods section). The top 30 genes in each layer were selected for further analysis by RNA *in situ* hybridization and immunohistochemistry.

We found several genes that were preferentially, or exclusively, expressed in neurons within a layer or sub-layer of the piriform cortex (Fig. 1b–d, Supplementary Fig. 1, Supplementary Table 1). For example, Reelin is enriched in densely packed neurons in layer IIa. Cux1 is selectively expressed in neurons of layers IIb and III, while Barhl1 expression marks a subpopulation of neurons confined to layer III. Using Tbr1 expression as a marker for excitatory neurons^31^, we found that the majority of Cux1^+^ and Barhl1^+^ cells were glutamatergic projection neurons. Similarly, the majority of the Reelin^+^ cells located in layer IIa were excitatory neurons. In contrast, most of the Reelin^+^ cells in layers I and III were GABAergic inhibitory neurons (Supplementary Fig. 2).

### Piriform projection neurons segregate into distinct layers

We next asked whether connectivity patterns of projection neurons could similarly segregate piriform neurons into layers and sub-layers. First, to identify potential piriform targets, we injected adeno-associated virus (AAV) expressing channelrhodopsin (ChR2)-eYFP^32^ into the piriform cortex. AAV-ChR2-eYFP infection of piriform neurons yields robust eYFP expression on cell somata and axon terminals, and can therefore serve as an anterograde tracer to identify brain regions innervated by piriform fibres. We detected strong YFP signals in several brain regions, including the OB, the AON, the OT, the lateral and medial PFC, the posteromedial and the posterolateral cortical amygdaloid nucleus, the agranular insular cortex and the lENT (Supplementary Fig. 3). We focused our analysis on piriform connections to the OB, the infralimbic subdivision of the medial PFC (IL-mPFC), the lENT and the posteromedial cortical amygdaloid nucleus (CoA). These four target areas are anatomically well separated, thus minimizing the possibility of cross contamination by neural tracer injections. We cannot exclude, however, that small amounts of tracer diffuse from the mPFC to the dorsal peduncular cortex and the dorsal tenia tecta, and from the posteromedial to the posterolateral cortical amygdaloid nucleus. The IL-mPFC and CoA-projecting subpopulations of piriform neurons we describe henceforth may therefore contain neurons projecting to more than one target area.

Next, we injected the retrograde neural tracer cholera toxin B subunit (CTB) coupled to green or red fluorophores into these potential piriform target areas (Supplementary Fig. 4) and assessed patterns of CTB labelling in the piriform cortex, at a rostro-caudal position 0.8–1.2 mm posterior to Bregma. These retrograde tracing experiments revealed a striking segregation of piriform neurons with distinct target specifications into layers and sub-layers (Fig. 1e–h). CTB injections into the OB resulted in the abundant labelling of neurons in layers IIb and III, but not in layers I and IIa (Fig. 1e). CTB injections into the IL-mPFC resulted in the labelling of neurons that were predominantly located in layer IIb, with only few cells in layers I and III. CTB-positive IL-mPFC-projecting neurons were absent from layer IIa (Fig. 1f). Piriform neurons projecting to the CoA were abundant in layer IIa, rarely observed in layers IIb and III, and excluded from layer I (Fig. 1g). Similarly, piriform neurons projecting to the lENT were predominantly found in layer IIa, and only few cells were located in layers I, IIb and III (Fig. 1h). These data indicate that piriform neurons projecting to different target areas segregate into distinct layers and sub-layers, and that piriform neurons within a layer or sub-layer can project to multiple target areas.

### Projection neurons exhibit distinct molecular identities

Gene expression analysis and retrograde tracing experiments reveal striking similarities in the segregation of piriform neurons into layers and sub-layers. This observation suggests that gene expression patterns may directly correlate with connectivity, that is, that the connectivity of individual piriform neurons can be inferred from their molecular identity. Thus, we next asked whether the layer-specific genes we have identified could provide information about the connectivity of piriform projection neurons, by combining retrograde tracing experiments with immunohistochemical analyses for layer-specific markers.

**Figure 2:**
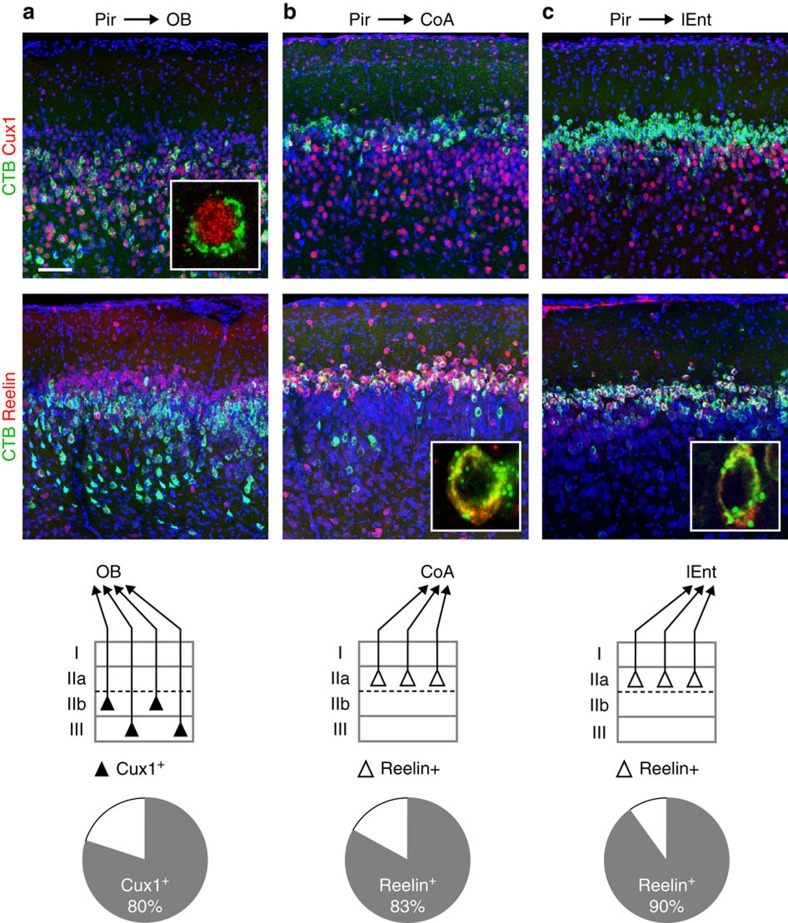
Genes delineate neurons with distinct connectivities. (**a**) Cux1 marks OB-projecting neurons. CTB-labelled cells are shown in green, immunohistochemistry for Cux1 or Reelin in red. Insert: a CTB^+^/Cux1^+^ cell at high magnification. Fraction of CTB-labelled cells expressing Cux1: 80±6%. OB-projecting neurons are Reelin^−^. Scale bar, 100 μm. (**b**) Reelin marks CoA-projecting neurons. Insert: a CTB^+^/Reelin^+^ cell at high magnification. Fraction of CTB-labelled cells expressing Reelin: 83±6%. CoA-projecting neurons are Cux1^−^. (**c**) Reelin also marks lENT-projecting neurons. Insert: a CTB^+^/Reelin^+^ cell at high magnification. Fraction of CTB-labelled cells expressing Reelin: 90±4%. lENT-projecting neurons are Cux1^−^. Fractions of co-labelled cells are mean±s.e.m. (see main text for details). Schemes represent the laminar distribution and the molecular identities of neurons with distinct connectivities.

A correlation of gene expression with connectivity is indeed readily apparent where territories delineated by layer-specific genes and output specificity coincide. For example, neurons projecting to the OB are present in layers IIb and III, and the majority of OB-projecting neurons are Cux1^+^ (mean=80%, s.e.m.=6, 999 Cux1^+^/CTB^+^ out of 1,250 CTB^+^ cells; *n* (number of mice)=4, Fig. 2a). In contrast, no Reelin^+^ OB-projecting neurons were observed. On the other hand, neurons projecting to the CoA and the lENT, which are predominantly present in layer IIa, are Cux1^−^, but Reelin^+^ (CoA: Reelin^+^ neurons: mean=83%, s.e.m.=6, 416 Reelin^+^/CTB^+^ out of 489 CTB^+^ cells; *n*=3. lENT: Reelin^+^ neurons: 90%, s.e.m.=4, 695 Reelin^+^/CTB^+^ out of 771 CTB^+^ cells; *n*=3, Fig. 2b, c). Furthermore, we observed that this link between the laminar position, molecular identity and connectivity of OB- and CoA-projecting neurons was maintained over a 2-mm distance along the rostro-caudal axis of the piriform cortex (Supplementary Fig. 5).

To confirm the observed segregation of Cux1^+^ OB-projecting and Reelin^+^ CoA-projecting neurons, we next used an intersectional viral tracing approach. We injected Cre-dependent AAV-flex-ChR2-eYFP into the piriform cortex, and we injected the retrogradely transported canine adenovirus 2 (CAV2)-Cre^33^,^34^ into the OB or the CoA (Supplementary Fig. 6). We found that the majority of OB-projecting neurons (54 out of 62 cells, *n*=5) were located in piriform layers IIb and III, while the majority of CoA-projecting neurons (41 out of 45 cells, *n*=7) were located in layer IIa. Furthermore, this experimental strategy allowed us to sparsely label OB- and CoA-projecting piriform neurons and reconstruct their morphologies. We found that OB-projecting neurons exhibited diverse morphologies characteristic of superficial and deep pyramidal cells, while CoA-projecting neurons exhibited semilunar cell-characteristic morphologies^29^,^35^.

**Figure 3:**
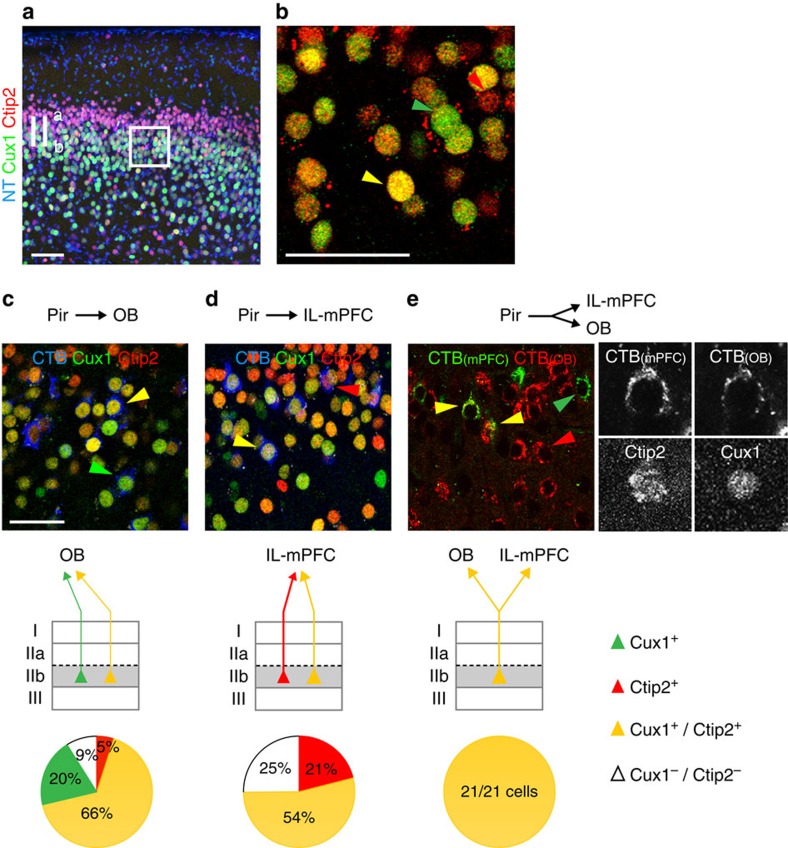
Gene expression patterns discriminate OB- and IL-mPFC-projecting neurons within layer IIb. (**a**) Ctip2 expression (in red) and Cux1 expression (in green) partially overlap in neurons in piriform layers IIb and III. Neurotrace counterstain in blue. Scale bar, 100 μm. (**b**) High magnification of piriform layer IIb (boxed area in **a**). The combinatorial expression of Cux1 and Ctip2 specifies three different subclasses of projection neurons in piriform layer IIb, Cux1^+^ (in green, green arrowhead), Ctip2^+^ (in red, red arrowhead), and Cux1^+^/Ctip2^+^ (in yellow, yellow arrowhead). Scale bar, 50 μm. (**c**) OB-projecting neurons in layer IIb are Cux1^+^/Ctip2^+^ (66±16%), or Cux1^+^/Ctip2^−^ (20±8%). Only 5±2% are Cux1^−^/Ctip2^+^. Scale bar, 50 μm. (**d**) IL-mPFC-projecting neurons in piriform layer IIb are Cux1^+^/Ctip2^+^ (54±12%), or Cux1^−^/Ctip2^+^ (21± 3%). No Cux1^+^/Ctip2^−^ IL-mPFC-projecting neurons were observed. (**e**) Injection of green CTB into the IL-mPFC, and red CTB into the OB reveals layer IIb cells projecting to both target areas (yellow). All double-projecting neurons (21 out of 21) are Cux1^+^/Ctip2^+^. Grey scale images show a Cux1^+^/Ctip2^+^, OB/IL-mPFC double-projecting neuron. Fractions of co-labeled cells are mean±s.e.m. (see main text for details). Schemes represent the laminar distribution and the molecular identities of neurons with distinct connectivities.

In contrast to the spatially non-overlapping populations of OB- and CoA-projecting neurons, CTB injections into the OB and IL-mPFC label overlapping subpopulations of neurons in layer IIb (Fig. 1e,f), suggesting that molecularly distinct neurons projecting to either target area could be intermingled within this layer. If the molecular identity of a neuron can indeed reveal its connectivity, we predict that the distinct subpopulations of layer IIb projection neurons can be discriminated based on gene expression profiles. To test this hypothesis, we combined CTB injections into the OB and the IL-mPFC with immunohistochemistry for Cux1 and Ctip2, two transcription factors that mark partially overlapping subpopulations of neurons in piriform layer IIb (Fig. 3a,b). We found that the majority of OB-projecting neurons in layer IIb were Cux1^+^ (mean=86%, s.e.m.=9, 410 Cux1^+^/CTB^+^ out of 479 CTB^+^ cells; *n*=3, Fig. 3c). Of these, 20% were Cux1^+^/Ctip2^−^, and 66% were Cux1^+^/Ctip2^+^. Only 5% of OB-projecting neurons were Cux1^−^/Ctip2^+^ (s.e.m.=2, 25 Ctip2^+^/CTB^+^ out of 479 CTB^+^ cells; *n*=3), while 9% of neurons expressed neither of the two genes. In contrast, 75% of IL-mPFC-projecting neurons were Ctip2^+^ (s.e.m.=14, 122 Ctip2^+^/CTB^+^ out of 137 CTB^+^ cells; *n*=4. 21% Ctip2^+^/Cux1^−^, 54% Ctip2^+^/Cux1^+^, Fig. 3d). Strikingly, no Cux1^+^/Ctip2^−^ IL-mPFC-projecting neurons were observed, while 25% of IL-mPFC-projecting neurons expressed neither of the two genes. These observations suggest that three distinct subclasses of layer IIb projection neurons can be discriminated based on their molecular identities: Cux1^+^/Ctip2^−^ cells, projecting to the OB; Cux1^−^/Ctip2^+^ cells, projecting to the IL-mPFC; and Cux1^+^/Ctip2^+^ neurons, projecting to both target areas. To directly test this prediction we injected CTB coupled to a red fluorophore into the OB, and CTB coupled to a green fluorophore into the IL-mPFC. Immunohistochemical analysis of cells labelled with the red and the green fluorophore, and therefore simultaneously projecting to both target areas, revealed that all double-projecting neuron indeed expressed both Cux1 and Ctip2 (total number of neurons=21; *n*=2, Fig. 3e).

While limitations in the efficiency of CTB uptake and the large number of potential molecular markers and piriform target areas preclude a complete, quantitative analysis of gene expression patterns and connectivity, our experiments suggest that the molecular identities of subclasses of piriform projection neurons can provide a predictive link with their connectivity, independent of whether they are segregated into distinct piriform layers or intermingled within a given layer.

### Neural identity is maintained in a scrambled cortex

We next asked if proper cortical lamination was a critical determinant of piriform output specificity. To address this question we examined piriform organization and connectivity in mice deficient for Reelin. Reelin is a secreted glycoprotein expressed by Cajal–Retzius cells during embryonic development. *Reeler* mice harbour a spontaneous mutation in the *Reelin* locus, which results in a loss of Reelin expression and severe defects in cortical lamination^36^,^37^,^38^.

**Figure 4:**
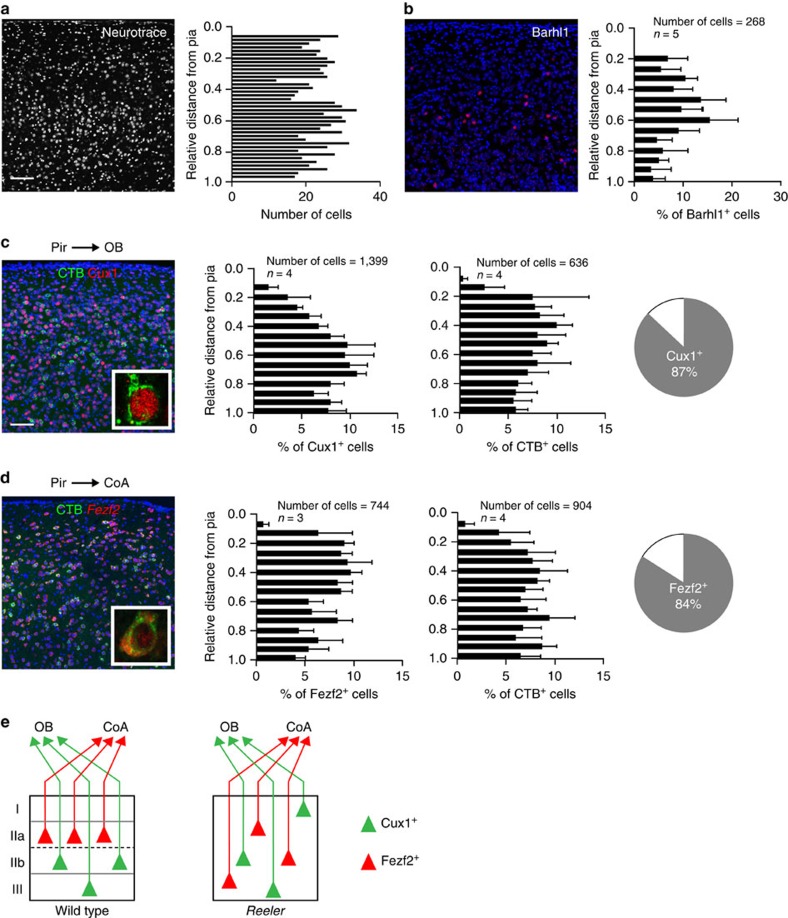
Molecular signatures of OB- and CoA-projecting neurons are independent of cortical layer positioning. In *reeler* mice, (**a**) cell density and (**b**) Barhl1^+^, (**c**) Cux1^+^ and (**d**) Fezf2^+^ neurons are evenly distributed across piriform cortex. Similarly, CTB injections into the OB (**c**) and the CoA (**d**) do not mark neurons that segregate into layers. (**c**) Cux1 marks OB-projecting neurons. Insert: a CTB^+^/Cux1^+^ cell at high magnification. Fraction of CTB-labelled neurons expressing Cux1: 87±1%. (**d**) *Fezf2* marks CoA-projecting neurons. Insert: a CTB^+^/*Fezf2*^+^ cell at high magnification. Fraction of CTB-labelled neurons expressing *Fezf2*: 84±2%. Fractions of co-labelled cells are mean±s.e.m. (see main text for details). Differential distributions of cells quantified in histograms are represented as mean±s.d. Scale bars, 100 μm. (**e**) Schematic representation of piriform projection neurons to OB and CoA in wild-type (left) and *reeler* (right) mice. Despite the scrambled positioning of the OB- and CoA- projecting neurons, their molecular identities are conserved in *reeler* mice.

We observed that Reelin deficiency similarly affects the formation of morphologically identifiable layers in the piriform cortex. Cell density did not significantly vary across the depth of piriform cortex (Fig. 4a). Layer-specific genes, including Cux1, Barhl1, Tle4, Foxp2 and Fezf2, were expressed in the piriform cortex of *reeler* mice. However, in sharp contrast to wild-type mice, neurons expressing these genes did not segregate into distinct layers but were instead intermingled and distributed throughout the depth of the piriform cortex (Fig. 4b–d, and Supplementary Fig. 7a). These observations suggest that Reelin is required for the proper layer organization of the piriform cortex. Furthermore, our experiments are consistent with previous reports suggesting that Reelin deficiency does not have a major effect on the molecular specification of cortical neurons^39^,^40^.

We next asked whether connections of piriform neurons with their downstream target areas were maintained in *reeler* mice. We focused on the OB and the CoA, as the anatomical organization of these two structures is largely unaffected by the *reeler* mutation^39^,^41^. We found that injections of CTB into the OB and CoA of *reeler* mice reliably labelled subpopulations of piriform neurons, and that the numbers of labelled neurons were similar to controls (Fig. 4c,d). However, while OB- and CoA-projecting neurons segregate into distinct, non-overlapping layers in wild-type mice, no such segregation was observed in *reeler* mice. OB- and CoA-projecting neurons were present throughout the depth of piriform cortex and could not be discriminated based on their position (Fig. 4c,d).

Can gene expression patterns reveal information about piriform neural connectivity when the positioning of neurons is scrambled? To address this question, we combined retrograde neural tracing with immunohistochemistry for Cux1, a marker of OB-projecting neurons, and RNA *in situ* hybridization for *Fezf2*. *Fezf2* is specifically expressed in piriform layer IIa cells and co-expressed with Reelin in greater than 85% of neurons (s.e.m.=2, 284 Reelin^+^/*Fezf2*^+^ out of 336 *Fezf2*^+^ cells, *n*=3, Supplementary Fig. 7b), thus serving for an alternative marker of CoA-projecting neurons in the absence of Reelin expression. We found that, similar to wild-type mice, the majority of OB-projecting neurons were Cux1^+^ (87%, s.e.m.=<1, 504 Cux1^+^/CTB^+^ out of 580 CTB^+^ cells; *n*=4, Fig. 4c, compared with 80% in wild-type, Fig. 2a), while no OB-projecting neurons were found to express *Fezf2*. On the other hand, the majority of CoA-projecting piriform neurons expressed *Fezf2* (84%, s.e.m.=2, 439 *Fezf2*^+^/CTB^+^ out of 514 CTB^+^ cells; *n*=3, Fig. 4d), but not Cux1. Therefore, the molecular identities of OB- and CoA-projecting piriform neurons are maintained in *reeler* mice and provide information about their connectivity, even in the absence of their spatial segregation into distinct, non-overlapping piriform layers.

## Discussion

We have identified genes expressed in subpopulations of piriform neurons that segregate into distinct layers and sub-layers of the piriform cortex. A strikingly similar segregation into layers and sub-layers is observed for piriform projection neurons connecting with different target areas. Our results suggest that the connectivity of piriform neurons can be inferred from their molecular identity. These molecular signatures of connectivity are maintained in Reelin-deficient mice, in which the laminar organization of the piriform cortex is lost and the positions of projection neurons are scrambled. Our data suggest that in the absence of positional information, the molecular identities of piriform neurons are sufficient to assure the specificity of piriform output connectivity.

We used laser-capture micro-dissection and RNA deep sequencing to identify candidate genes enriched in individual piriform layers. Interestingly, transcriptome analysis for genes enriched in layer I produced many mRNAs encoding synaptic proteins, such as scaffolding proteins, signalling molecules and receptors (Supplementary Table 1, and data not shown). Piriform layer I is primarily comprised of the axon terminals of OB mitral cells, the dendrites of layer II/III neurons and a sparse population of interneurons. The identification of synaptic proteins suggests that our analysis detects transcripts locally translated in the neuropil, consistent with results obtained by the transcriptome analysis of synaptic neuropil of the CA1 area of the rat hippocampus^42^. In contrast, many genes enriched in layers II and III are transcription factors and axon guidance molecules. These layer-specific piriform genes exhibit various degrees of selectivity. Cux1, for example, is expressed in a large population of cells. Cux1^+^ neurons are molecularly and morphologically heterogeneous and include glutamatergic and GABAergic neurons. In contrast, Tle4, Barhl1 and Foxp2 are expressed in sparse subpopulations of excitatory projection neurons that are strictly confined to layer III. Interestingly, Reelin expression is observed in two distinct and spatially segregated subpopulations of piriform neurons. Similar to the neocortex^43^, we find that Reelin is expressed in sparse populations of predominantly GABAergic interneurons in piriform layers I and III. In addition, we identify densely packed Reelin^+^ neurons in layer IIa. Reelin^+^ layer IIa cells express the transcription factors Tbr1 and Fezf2, and exhibit the characteristic morphologies of semilunar cells.

The gene expression patterns we describe for the adult piriform cortex likely reflect the outcome of developmental genetic programs that control the differentiation, maturation and connectivity of distinct subclasses of piriform neurons. Interestingly, our data indicate that these transcriptional programs are strikingly different from those observed in the neocortex. In the neocortex, Cux1 marks the superficial layers II/III, while Fezf2 is exclusively expressed in neurons of the deep layers V and VI. Notably, this organization is reversed in the piriform cortex: Fezf2 expression is restricted to superficial cells in layer IIa, while Cux1 is expressed in deeper portions of layers IIb and III. Furthermore, Cux1 and Ctip2, a marker of the deep neocortical layers V and VI^44^, are expressed in mutually exclusive subpopulations of neocortical neurons. In sharp contrast, Cux1 and Ctip2 are co-expressed in about 30% of piriform layer IIb cells. These observations challenge the view of piriform cortex as a simple, ancestral cortical structure. A comparative analysis of the genetic programs that control neocortical and piriform neural specification may provide important new insights into the evolution of cortical neural circuits. Moreover, the genes we have identified will provide genetic access to distinct subclasses of piriform neurons, thus facilitating the analysis of their functions in the routing of odour information in the olfactory system.

In addition to the laminar distribution of piriform projection neurons, rostro-caudal patterns of organization are also likely to reflect the specificity of piriform connections. While we observe that the molecular identities of OB- and CoA-projecting piriform neurons are maintained along the rostro-caudal axis, a recent study has demonstrated that piriform projections to two other subdivisions of the PFC, the agranular insular cortex and the lateral orbitofrontal cortex, segregate along the rostro-caudal axis of the piriform cortex^14^. It will be interesting to test whether this graded connectivity is matched by the graded expression of genes that specify these subclasses of projection neurons.

Is the correct positioning of neurons into distinct cortical layers required for the specification of piriform neurons and the establishment of connections with appropriate downstream targets? We observed that in *reeler* mice, the laminar organization of the piriform cortex is lost, and the positions of piriform neurons are scrambled. Loss of Reelin, however, does not appear to have any major impact on the specification of piriform neural subtypes. For example, we did not observe any significant differences in the absolute or relative numbers of Ctip2-, Cux1-, Tle4-, Barhl1-, Foxp2- and Fezf2-expressing neurons. These findings are consistent with recent studies on neural specification in the neocortex and amygdala of *reeler* mice^39^,^40^. Furthermore, connections of piriform neurons with many piriform target areas appear to be at least partially maintained; our antero- and retrograde tracing experiments suggest that piriform neurons in *reeler* mice still project to all major target areas (data not shown). Importantly, molecular signatures of connectivity are maintained in *reeler* mice despite the scrambling of neural position. In wild-type mice, CoA- and OB-projecting neurons segregate into layers IIa, and IIb and III, respectively. In Reeler mutant mice, CoA- and OB-projecting neurons are distributed throughout the depth of piriform cortex in an intermingled fashion. However, CoA- and OB-projecting neurons can easily be discriminated based on the mutually exclusive expression of Fezf2 and Cux1.

Our finding, that a predictive link between neural identity and connectivity is maintained despite the scrambling of neural position, is reminiscent of motor neuron organization and connectivity in the spinal cord. The clustering of motor neurons into discrete motor pools critically depends on cadherin–catenin signalling, and the functional inactivation of β- and γ-catenin scrambles motor neuron position. β- and γ-catenin inactivation, however, does not perturb motor neuron identity and connectivity^45^. Therefore, for both the punctuate design of the neuromuscular map and the laminar organization of the olfactory cortex, the specificity of neural circuit output primarily depends on the molecular identity of the neurons rather than the precise positioning of neurons into clusters and layers.

## Methods

### Mice

Adult (8- to 12-week-old) male C57BL/6 wild-type mice, and *reeler* mice^36^ (obtained from the Jackson Laboratory, stock number 000235) were used in this study. Mice were housed at the animal facility at the CIRB/College de France. All experiments were performed according to European and French National institutional animal care guidelines (protocol number B750512/00615.02).

### Laser-capture micro-dissection and RNA deep sequencing

Brain sections were obtained from 10 week-old C57BL/6 mice. Coronal sections (30 μm thick) were cut at 0.8–1.2 mm posterior to Bregma using a Microm HM560 cryostat and mounted on PET-membrane slides (Leica). The three layers of piriform cortex were dissected using the Laser Micro Dissector (Leica LMD 7000) according to the manufacturer's recommendations. RNA was extracted from the collected tissue using the RNeasy Micro Kit (Qiagen). RNA amplification (Ovation RNA-Seq system, NuGEN), library preparation and Illumina sequencing were performed at the Ecole Normale Superieure Genomic Platform (Paris, France). Libraries were prepared using the strand non-specific RNA-Seq library preparation TruSeq RNA Sample Prep Kits v2 (Illumina). Libraries were multiplexed by three on two single-flowcell lanes and subjected to 50 bp paired-end read sequencing on a HiSeq 1,500 device (Illumina). Reads were aligned against the Mus musculus reference sequence (mm10—UCSC) using Bowtie mapper (version 0.12.9). Statistical treatments and differential analyses were performed using DESeq 1.8.3. Additional analysis was performed to extract gene sets by using Independent Component Analysis with parameters set as described in ref. 30. For each layer comparison, genes were sorted based on their expression log ratio and used as ranked gene list for GSEA analysis. Independent Component Analysis gene sets were then tested for their behaviour on these ranked gene lists.

### RNA *in situ* hybridization

Mouse brains were dissected and rinsed in ice-cold PBS, embedded in OCT (Tissue-Tek), snap frozen in liquid nitrogen and stored at −80 °C. Coronal brain sections (16 μm thick) were prepared using a cryostat. Two-colour RNA hybridization with DIG- and FITC-labelled probes was performed at 60–65 °C overnight. The RNA probes were labelled via *in vitro* transcription with T3, T7 or SP6 RNA polymerase (Roche and Promega) in the presence of digoxigenin (DIG)- or fluorescein isothiocynate (FITC)-labelled dUTP (Roche). Linearized plasmids (*Fezf2, Btbd3*, FANTOM Consortium; *Rorb*, gift from S.J. Chou) or PCR products obtained from piriform complementary DNA (*Prdm8*, *Zic1*, *Npcd*) were used as templates. Complementary DNA was prepared (SuperScriptVILO cDNA Synthesis system, Invitrogen) from 2 μg of total RNA, extracted from adult piriform cortex using Trizol (Invitrogen). PCR primers: *Prdm8* fw- 5′- TGCCAAGGCTGTCCAACAGTGTCTGAC -3′; rev- 5′- GGAACCGCCTCTTGCCTTTGCAGC -3′. *Zic1* fw- 5′- GATCAGAACAAGCTCTCTGGGGCTTC -3′; rev- 5′- CCAAGATCCACTAAAGCTGGCACAGG -3′. *Npcd* fw- 5′- GATCAACGACAAGGTGGCCCAGCTG -3′; rev-5′- CGAGGTAGGCCTGGAGCATAGGG -3′. First round PCR amplification protocol: 3 min at 94 °C, 12 cycles of 30 s 94 °C, 40 s at 48 °C and 5 min at 72 °C.

For the second round PCR amplification, the T7 promoter sequence was added to the reverse primers: *Prdm8* fw- 5′- ACATCCCAGAGAACGCCATATTCGGTC -3′; rev- 5′- TTCCAGTTCCCGAGCCAAGTTGTGG -3′. *Zic1* fw- 5′- GCTCTCTGGGGCTTCAGCTTTTC -3′; rev- 5′- CAGTTCTGGAAACTAAAGTGTACATACGAG -3′. *Npcd* fw- 5′- CCTGAAAGACAGCAACTGGCACC -3′; rev- 5′- GTCAAAGGCTACCTCTACCCACG -3′. Second round PCR amplification protocol: 3 min at 94 °C, 35 cycles of 30 s 94 °C, 40 s at 60 °C, and 3 min at 72 °C and 10 min at 72 °C. The DIG probes were detected with sheep anti-DIG antibody (1:1,000 dilution) conjugated to alkaline phosphatase (1:1,000 dilution, Roche) and visualized using BCIP/NBT (Roche). The fluorescent signal of FITC and DIG probes were detected with sheep anti-FITC or anti-DIG antibodies conjugated to horseradish peroxidase POD (Roche) and visualized using FITC or Cy3 tyramide (Perkin-Elmer). Neurotracer (Molecular Probes) was used as a counterstain. Images were acquired with a Nikon 90i widefield microscope and a Leica SP5 confocal microscope.

### Immunohistochemistry

Mice were deeply anaesthetised with pentobarbital and transcardially perfused with 10 ml of PBS, followed by 10 ml of 4% paraformaldehyde. Brains were post-fixed for 5 h in 4% paraformaldehyde at 4 °C. Coronal sections (200 μm thick) were prepared using a vibrating-blade microtome (Microm Microtech). Sections were rinsed in PBS and permeabilized in PBS/0.1% Triton X-100 for 1 h, and blocked in PBS/0.1% Triton X-100/2% heat-inactivated horse serum (Sigma) for 1 h. After incubation with primary antibodies at 4 °C overnight, sections were rinsed in PBS/0.1% Triton X-100, three times for 15 min at room temperature, blocked in PBS/0.1% Triton X-100/2% heat-inactivated horse serum for 1 h and incubated with secondary antibodies overnight at 4 °C. The following antibodies were used at the indicated dilutions: rabbit anti-Tbr1 1:500 (Abcam ab31940), rat anti-Ctip2 1:100 (Abcam AB18465), mouse anti-Reelin 1:500 (Millipore MAB5364), rabbit anti-Cux1 1:50 (Santa Cruz sc-13024), goat anti-Tle4 1:50 (Santa Cruz sc-13377), rabbit anti-Barhl1 1:500 (Sigma HPA 004809), goat anti-Foxp2 1:250 (Santa Cruz sc-21069), chicken anti-GFP 1:1,000 (Abcam ab13970). Appropriate secondary antibodies (1:1,000) conjugated to Cy3 (Jackson Labs) or Alexa 405, 488, 568 or 647 (Molecular Probes) were incubated together with Neurotrace counterstain (1:500, Invitrogen). Sections were mounted on SuperFrost Plus (Menzel-Gläser) microscope slides in Fluorescent Vectashield Mounting Medium (Vector). Images were acquired with a Leica SP5 confocal microscope

### Anterograde and retrograde tracing

Mice were anaesthetised with ketamine/xylazine (100 mg kg^−1^ per 10 mg kg^−1^, Sigma-Aldrich) and a small craniotomy was made above the injection site. For anterograde neural tracing experiments, 0.7 μl of AAV5.hSyn.hChR2(H134R)-eYFP.WPRE.hGHpA was stereotaxically into the piriform cortex. For intersectional viral tracing experiments, 0.4 μl of AAV1.EF1α.DIO.hChR2(H134R)-eYFP.WPRE.hGHpA was injected into the piriform cortex, and 0.4 μl of canine adenovirus (CAV)-2-Cre^33^,^46^ was injected into the OB or the CoA. For labelling GABAergic interneurons, 0.7 μl of AAV1.EF1α.DIO.eGFP.WPRE.hGHpA was injected into the piriform cortex of Gad2-Cre transgenic mice^47^. Virus was injected using a glass pipette with a 10–20 μm tip diameter. AAVs were obtained from the UNC Vector Core (University of North Carolina at Chapel Hill). CAV2-Cre was obtained from the Montpellier Vector Platform (PVM). For retrograde neural tracing experiments, 2 μl of 1% CTB conjugated to Alexa 488 and 594 (Molecular Probes) was injected at 0.20 μl min^−1^ using a Hamilton 1701 syringe with a 33GA needle, controlled by a nanoliter infusion pump (Kd-Scientific). The following coordinates, based on the Paxinos and Franklin Mouse Brain Atlas were used: piriform cortex: anterior-posterior (AP) -0.60, medio-lateral (ML) 3.98, dorso-ventral (DV) 4.00; OB: AP and ML coordinates were determined visually, DV 0.50; medial PFC (including the prelimbic (PrL), infralimbic (IL), and cingulate (Cg) cortex): AP +1.41, ML 0.20, DV 1.70; posteromedial cortical amygdaloid nucleus: AP −2.90, ML 2.70, DV 4.55; lENT: AP −3.80, ML 3.75, DV 2.80.

### Quantification of gene expression and retrograde tracing

All image processing and quantification was performed in ImageJ and Adobe Photoshop CS5. Immunohistochemical analysis of piriform neurons was obtained from sections at 0.8–1.2 mm posterior to Bregma, except for Supplementary Fig. 5. For each mouse, cells in 4 fields of view (738 × 738 μm) were counted unless indicated otherwise. The depth of piriform cortex was measured based on histological reference points defined in the Paxinos and Franklin Mouse Brain Atlas, and the relative position of cells across the depth of piriform cortex was determined by measuring the distance of a cell from the pia, divided by the depth of piriform cortex. A given field of view was divided into 15 bins, and the fraction of cells in each bin was calculated as the total number of cells in each bin, divided by the total number of cells in the field of view. The fraction of projecting neurons expressing a given gene was calculated as the number of CTB-labelled cells expressing a given gene, divided by the total number of CTB-labelled cells. The morphologies of ChR2-eYFP-expressing cells were reconstructed from high resolution confocal images (12 bit grayscale, *x*, *y*: 0.6 μm/pixel, *z* step size 0.5 μm), using the ‘Simple Neurite Tracer' Image J plug-in^48^. Statistical analyses were performed using GraphPad Prism 6 software.

### Data availability

The RNA sequencing data and raw fastq files have been deposited in NCBI's Gene Expression Omnibus^49^ and are accessible through the GEO repository (www.ncbi.nlm.nih.gov/geo/query/acc.cgi?acc=GSE70800) under the accession number GSE70800.

## Additional information

**How to cite this article:** Diodato, A. *et al*. Molecular signatures of neural connectivity in the olfactory cortex. *Nat. Commun.* 7:12238 doi: 10.1038/ncomms12238 (2016).

## Supplementary Material

Supplementary Information Supplementary Figures 1-7 and Supplementary Table 1
