# Supplementary material for: Molecular signatures of neural connectivity in the olfactory cortex
Source: Nat Commun. 2016 Jul 18;7:12238. doi: 10.1038/ncomms12238 (PMC4960301; doi:10.1038/ncomms12238)
Supplement: Supplementary Information — Supplementary Figures 1-7 and Supplementary Table 1 [file ncomms12238-s1.pdf]

Supplementary Figure 1

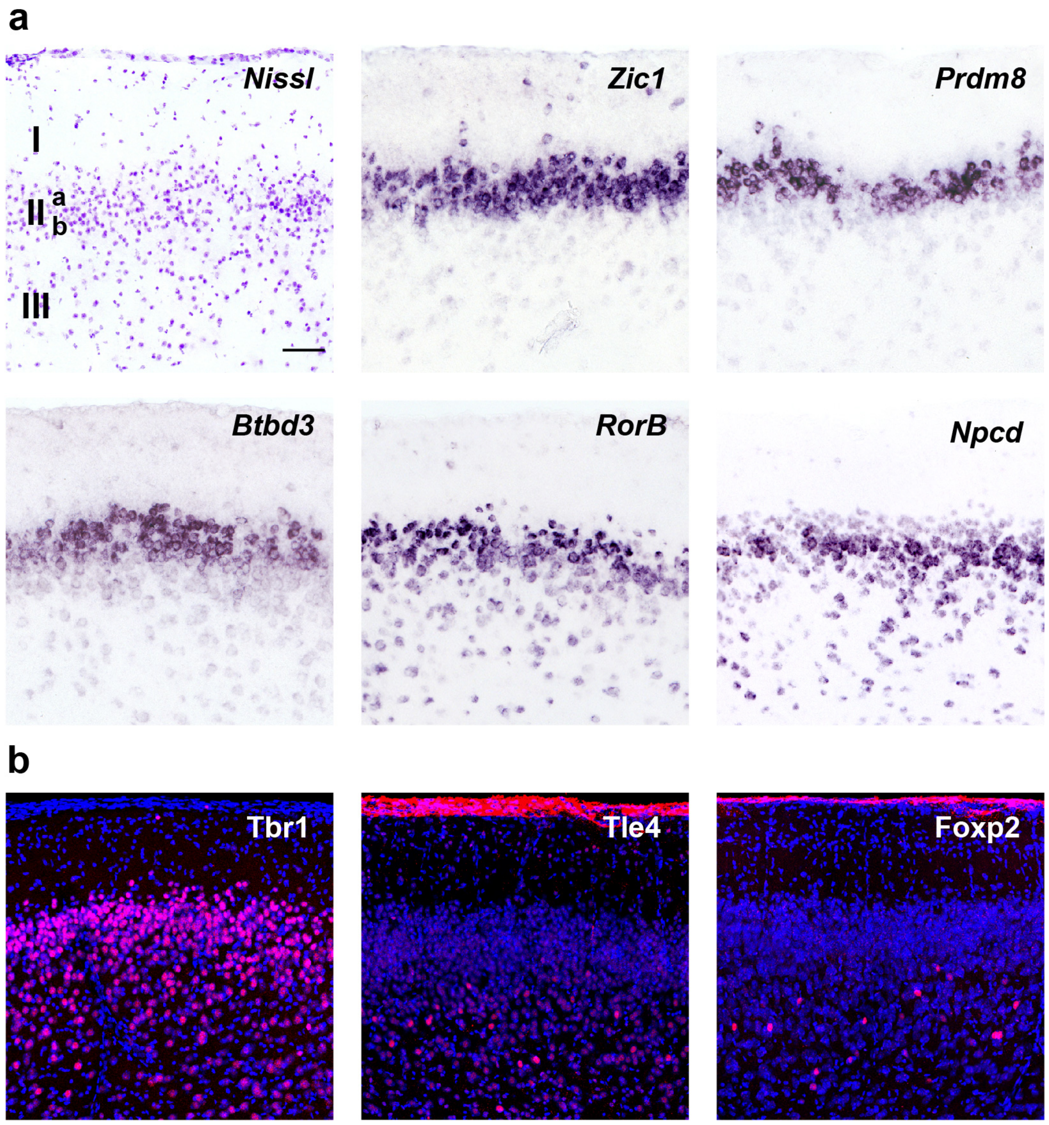

Supplementary Figure 1. Genes differentially expressed across piriform layers.
(**a**) *In situ* hybridization of genes differentially expressed across piriform layers. Nissle staining (top left) is shown as a reference for cell density. *Zic1* is expressed in neurons in layer II, and in a sparse subpopulation of neurons in layer I. *Prdm8* and *Btbd3* mark neurons in layer II. *RorB* is expressed in neurons in layer IIa, and in subpopulations of neurons in layers IIb and III. *Npcd* marks neurons in layer IIb and III. Scale bar = 100µm.
(**b**) Immunohistochemical detection of proteins differentially expressed across piriform layers. Tbr1 is a marker for cortical projection neurons and is broadly expressed in layers II and III. Tle4 and Foxp2 mark sparse subpopulations of neurons in layer III.

Supplementary Figure 2

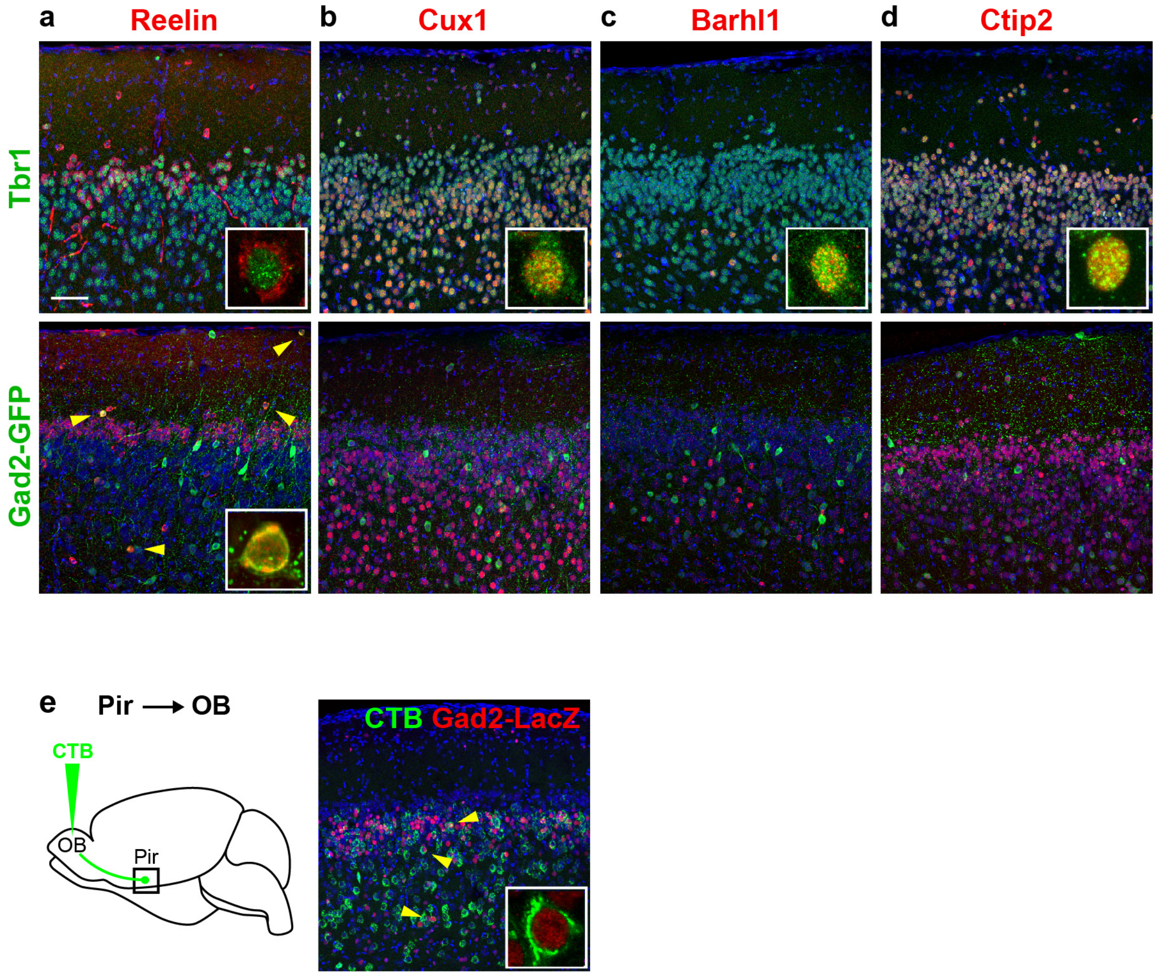

Supplementary Figure 2. Reelin+ cells in piriform layer IIa, and piriform Cux1+, Barhl1+, and Ctip+ cells are glutamatergic projection neurons.

(**a,** top panel) The majority of Reelin+ cell in piriform layer IIa express Tbr1+, a marker for glutamatergic projection neurons (>99%, 742 Tbr1+/Reelin+ out of 743 Reelin+ cells). Insert: a Reelin+/Tbr1+ cell at high magnification. In contrast, only one third of the Reelin+ cells in layers I and III are Tbr1+ (layer I: 32%, 15 out of 47 cells, layer III: 32%, 6 out of 19 cells).

(**a**, bottom panel) Conditional expression of GFP in Gad2-cre transgenic mice, via infection with AAV-flex-GFP, identifies GABAergic Reelin+ cells in layers I and III (yellow arrowheads), but not in layer IIa (layer I: 56%, 14 out of 25 cells, layer IIa: 1%, 5 out of 603 cells, layer III: 63%, 17 out of 27 cells). Scale bar = 100μm.

(**b**) The majority of of Cux1+ piriform neurons are Tbr1+ (97%, 709 out of 730 cells) (top). Insert: a Cux1+/Tbr1+ cell at high magnification. 6% (37 out of 606 cells) of Cux1+ cell are Gad2-cre/GFP+ (bottom).

(**c**) The majority of of Barhl1+ piriform neurons are Tbr1+ (95%, 107 out of 113 cells) (top). Insert: a Barhl1+/Tbr1+ cell at high magnification. 2% (3 out of 154 cells) of Barhl1+ cell are Gad2-cre/GFP+ (bottom).

(**d**) The majority of of Ctip2+ piriform neurons are Tbr1+ (95%, 1197 out of 1259 cells) (top). Insert: a Ctip2+/Tbr1+ cell at high magnification. 3% (19 out of 631 cells) of Ctip2+ cell are Gad2-cre/GFP+ (bottom).

(**e**) Identification of putative GABAergic piriform neurons projecting to the OB. CTB injections into the OB mark (in green) show putative GABAergic neurons, identified by the expression of the nuclear lacZ reporter (in red) in Gad2-cre; ROSA-lox-stop-lox-nlacZ transgenic mice (8%, 40 nlacZ+/CTB+ out of 522 CTB+ cells). For all co-labeling experiment, piriform neurons on three histological sections from two mice were counted.

Supplementary Figure 3

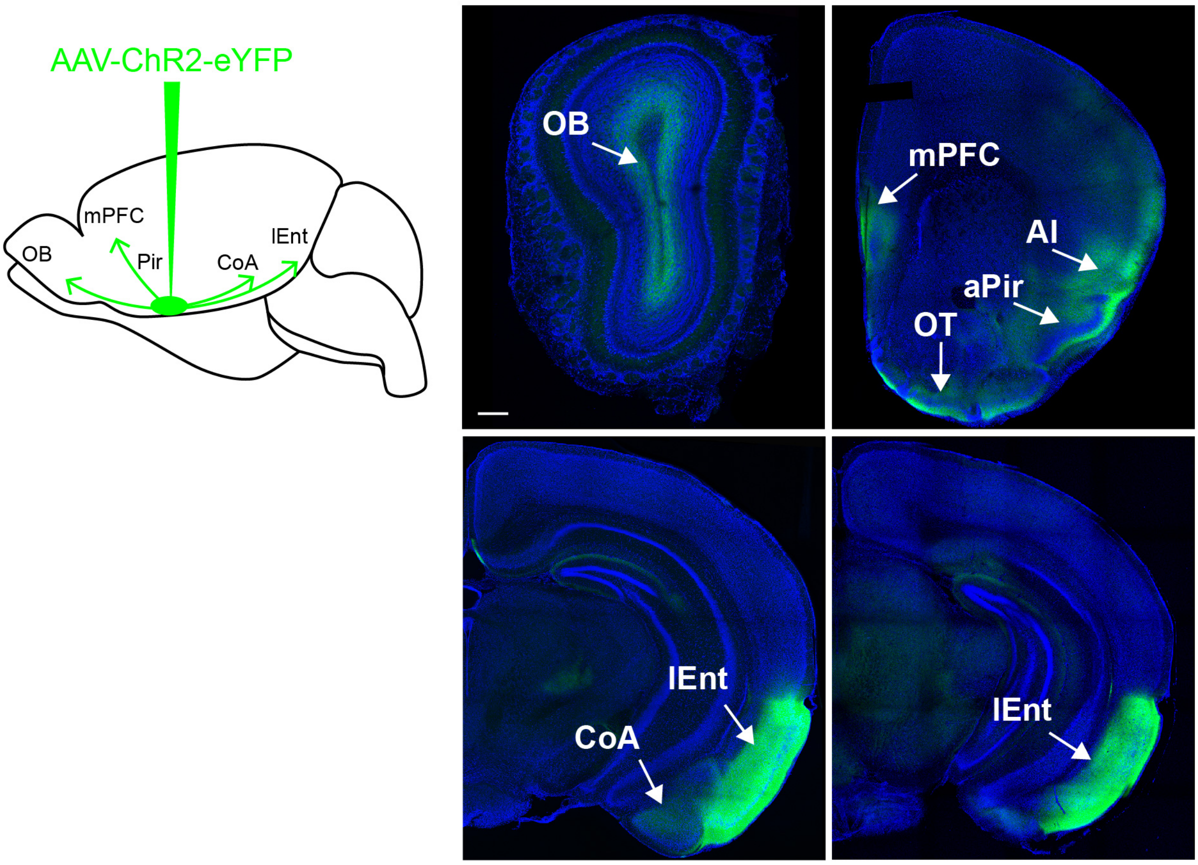

Supplementary Figure 3. Anterograde tracing of piriform outputs.
Identification of piriform target regions using anterograde neural tracing. Schematic representation of the AAV-ChR2-eYFP injection site and piriform target areas. eYFP expression identifies piriform projections to the olfactory bulb (OB), the infralimbic subdivision of the medial prefrontal cortex (mPFC), olfactory tubercle (OT), anterior piriform cortex (aPir), agranular insular cortex (AI), posterior-medial cortical amygdala (CoA), and the lateral entorhinal cortex (lENT). Scale bar = 500µm.

Supplementary Figure 4

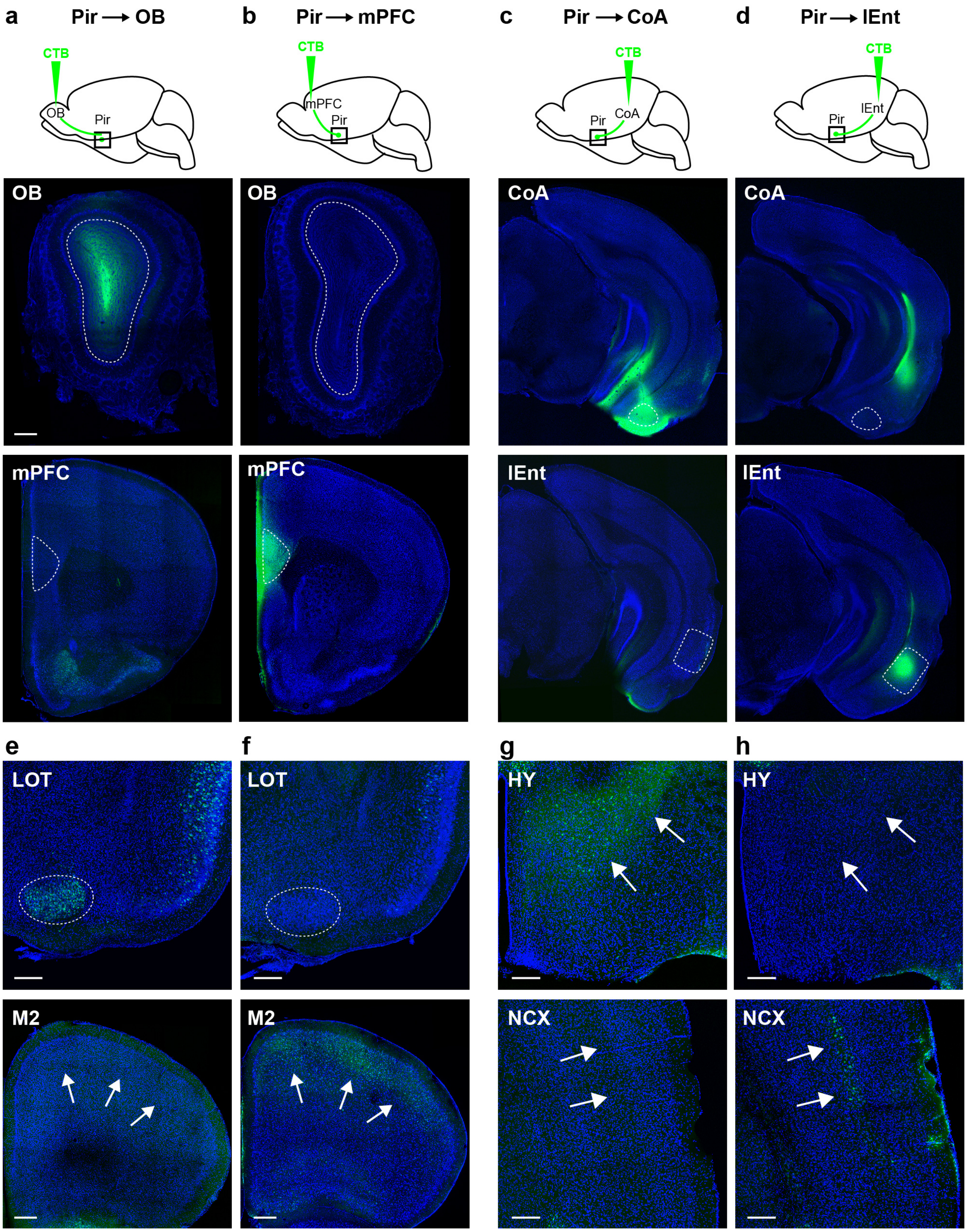

Supplementary Figure 4. Specificity of CTB injection sites.

(**a**-**d**, top panels) Representative examples of CTB injection sites into the (a) olfactory bulb (OB), (b) medial prefrontal cortex (mPFC), (c) posteromedial cortical amygdaloid nucleus (CoA), and (d) lateral entorhinal cortex (lENT). (a, b, bottom panel) CTB diffusion into the mPFC is not observed in the OB-injected brain. Conversely, CTB diffusion into the OB is not observed in the mPFC-injected brain. (c, d, bottom panel) CTB diffusion into the lENT is not observed in the CoA-injected brain. CTB diffusion into the CoA is not observed in the lENT-injected brain.

(**e**-**h**) CTB injections into the 4 piriform target areas shown in (a-d) mark distinct, non-overlapping populations of neurons in other brain areas. (e, f) CTB injections into the OB mark neurons in the nucleus of the lateral olfactory tract (LOT), but not in secondary motor cortex (M2). Conversely, CTB injections into the mPFC mark neurons in M2 but not in the LOT. (g, h) CTB injections into the CoA mark neurons in the hypothalamus (HY), but not in layer 5 of secondary somatosensory cortex (S2). CTB injections into the lENT mark neurons in S2 but not in the HY. Scale bars = 500µm.

Supplementary Figure 5

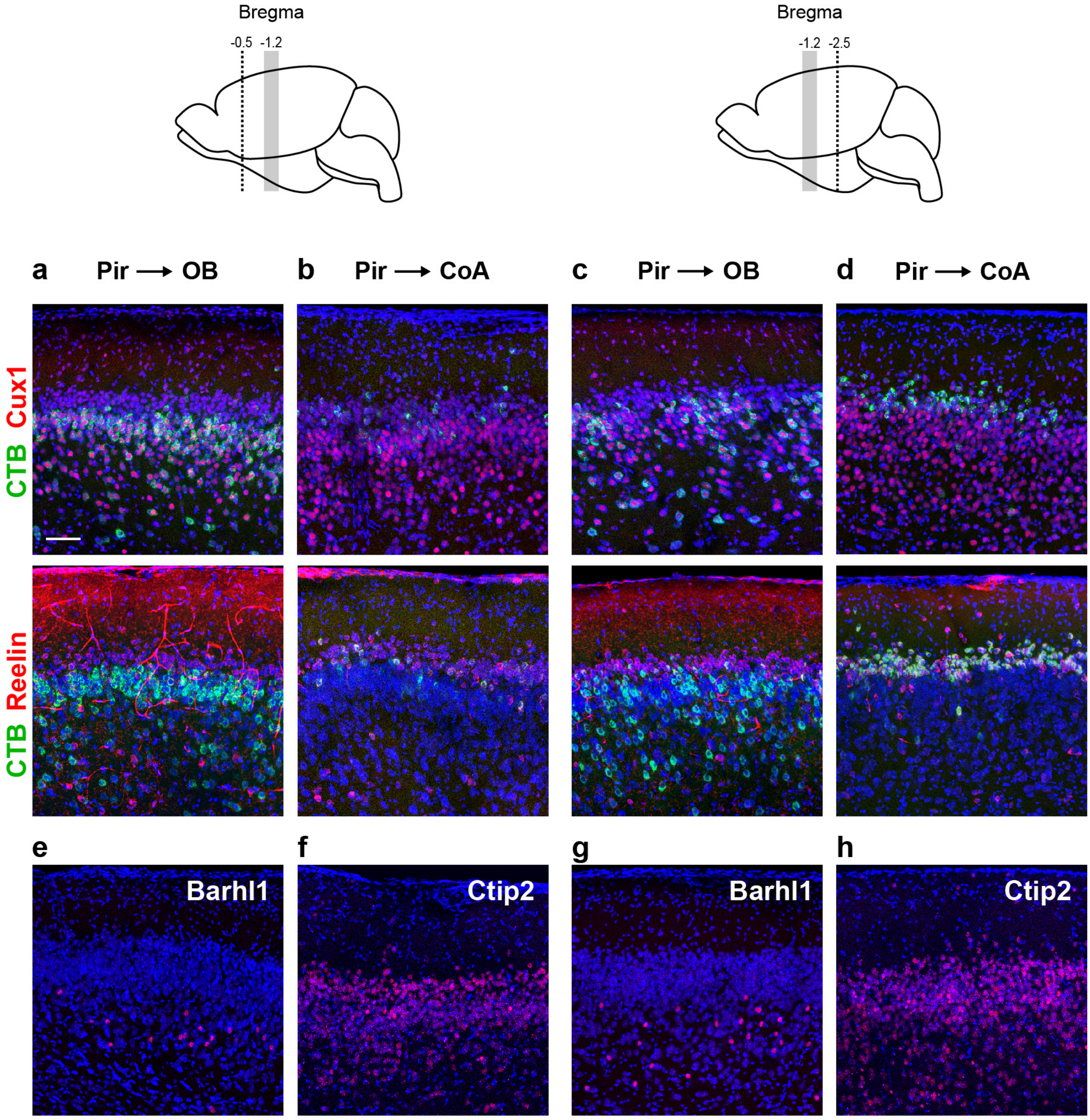

Supplementary Figure 5. Molecular signatures of connectivity are maintained along the rostro-caudal axis of piriform cortex.

(**a**, **b**) Representative examples of histological sections through the piriform cortex at 0.5 mm posterior to Bregma. Scale bar = 100µm.

(**c**, **d**) Representative examples of a histological section through the piriform cortex at 2.5 mm posterior to Bregma. The majority of OB-projecting neurons are located in piriform layers IIb and III, and are Cux1+/Reelin−. In contrast, the majority of CoA-projecting neurons are located in piriform layer IIa, and are Cux1−/Reelin+.

(**e** - **h**) The laminar distribution of Barhl1+ neurons (layer III) and Ctip2+ neurons (layers II and III) is maintained along the rostro-caudal axis of piriform cortex.

Supplementary Figure 6

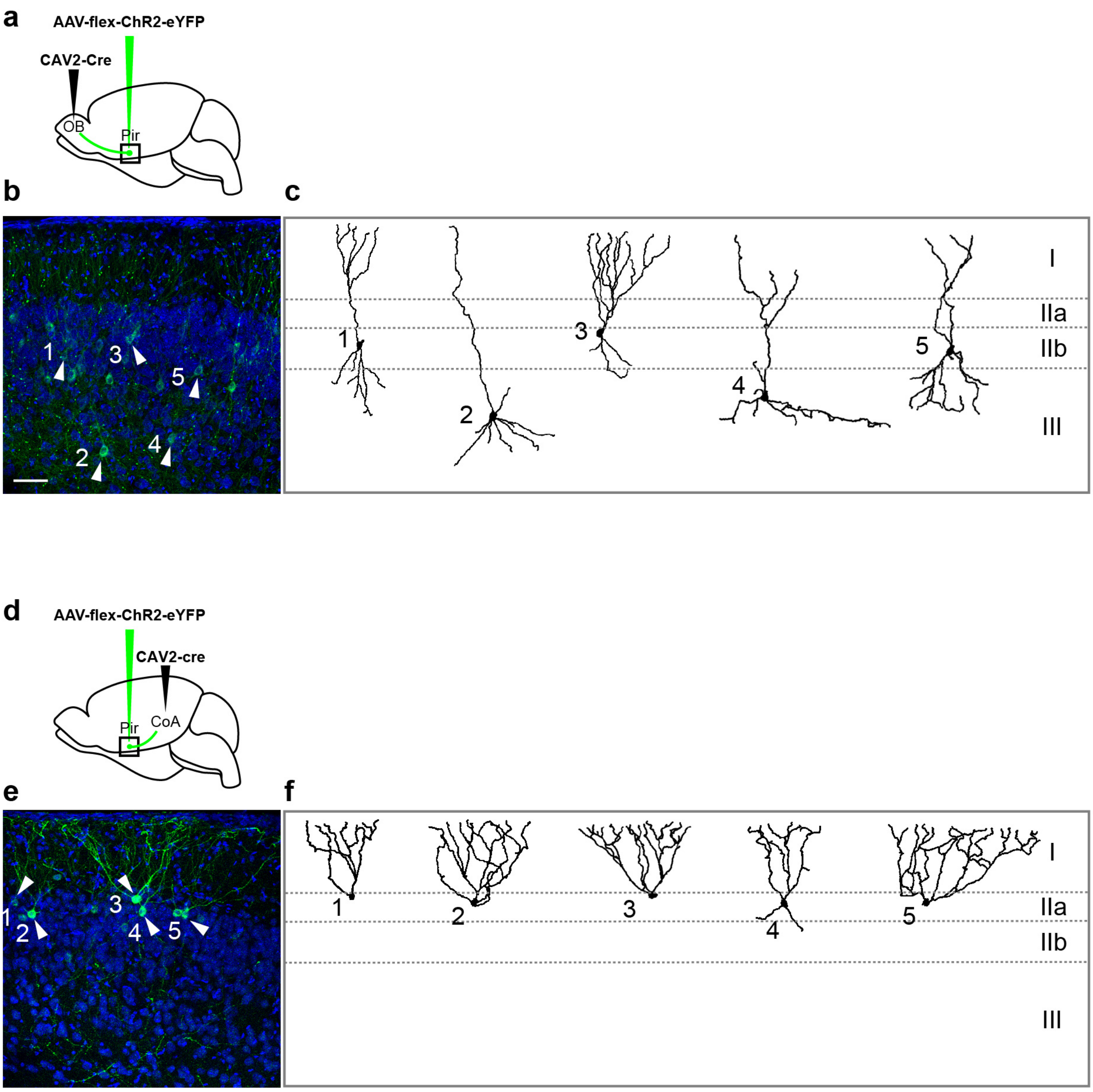

Supplementary Figure 6. Viral tracing of OB- and CoA-projecting piriform neurons.
(**a**-**c**) OB-projecting neurons exhibit diverse morphologies of superficial and deep pyramidal cells. (a) Scheme of virus injections. CAV2-Cre was injected into the OB, Cre-dependent AAV-flex-ChR2-eYFP was injected into the piriform cortex. (b) Immunohistochemical detection of ChR2-eYFP+ OB-projecting neurons (in green), Neuro-trace in blue. Scale bar = 100μm. (c) Morphologies of the 5 cells indicated by arrowheads in (b).
(**d**-**f**) CoA-projecting neurons exhibit the characteristic morphologies of semilunar cells. (d) Scheme of virus injections. CAV2-Cre was injected into the CoA, Cre-dependent AAV-flex-ChR2-eYFP was injected into the piriform cortex. (e) Immunohistochemical detection of ChR2-eYFP+ CoA-projecting neurons (in green), Neuro-trace in blue. (f) Morphologies of the 5 cells indicated by arrowheads in (e).

Supplementary Figure 7

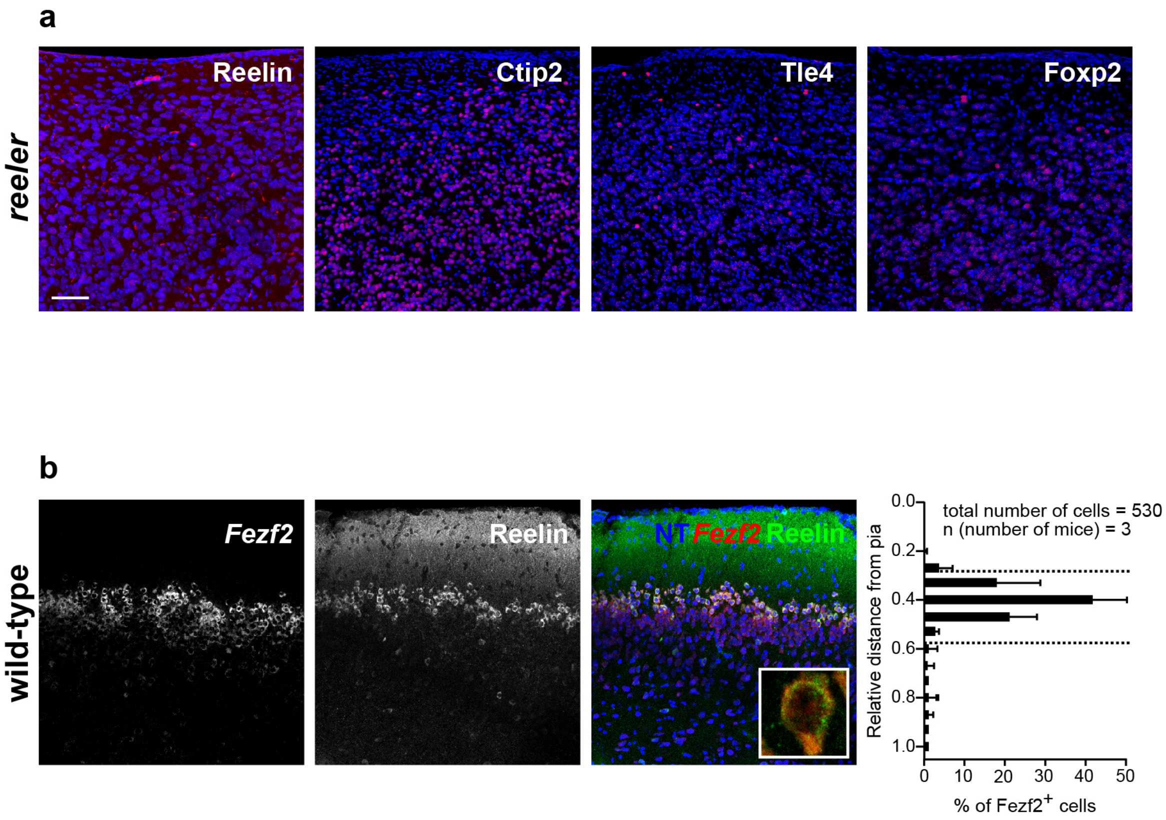

Supplementary Figure 7. Fezf2 and Reelin are co-expressed in layer IIa of piriform cortex.
(**a**) Immunohistochemical detection of Ctip2, Tle4 and Foxp2 in the piriform cortex of reeler mice. Ctip2, Tle4 and Foxp2 are expressed in subpopulations of piriform neurons. Neurons expressing these proteins are intermingled and distributed throughout the depth of the piriform cortex, and do not segregate into distinct piriform layers. Note the lack of Reelin expression in *reeler* mice. Scale bar = 100µm.
(**b**) Co-expression of *Fezf2* (*in situ* hybridization) and Reelin (immunohistochemistry) in piriform layer IIa cells in wild-type mice (insert: example of a *Fezf2*+/Reelin+ cell at high magnification). Neurotrace counterstain in blue. Quantification of the distribution of Fezf2-expressing neurons across the depth of piriform cortex in wild-type mice. Data are represented as mean ± SD.

	Gene name	Description	EnsemblGeneID	Fold increase	Histology	Result
**Layer I**	Slc13a3	solute carrier family 13, member 3	ENSMUST00000029208	20 x (LII)	-	-
	Lrp4	low density lipoprotein receptor-related protein 4	ENSMUST00000028689	15 x (LII)	ISH	enriched in LI and LIIa
	Fgfr2	fibroblast growth factor receptor 2	ENSMUST00000120187	13 x (LII)	ISH	non-specific signal
	Zic1	zinc finger protein of the cerebellum 1	ENSMUST00000034927	12 x (LIII)	ISH	enriched in LI and LII
	Prrx1	paired related homeobox 1	ENSMUST00000075805	10 x (LII)	ISH	non-specific signal
	Mical1	microtubule associated monooxygenase, calponin and LIM domain containing 1	ENSMUST00000099934	10 x (LIII)	-	-
	Vamp5	vesicle-associated membrane protein 5	ENSMUST00000101285	10 x (LIII)	-	-
	Slc22a8	solute carrier family 22 (organic anion transporter), member 8	ENSMUST00000010251	9.0 x (LI)	-	-
	Id4	inhibitor of DNA binding 4	ENSMUST00000021810	8.3 x (LII)	IHC	non-specific signal
	Cacna1s	calcium channel, voltage-dependent, L type, alpha 1S subunit	ENSMUST00000161865	6.7 x (LIII)	-	-
	Robo4	roundabout homolog 4 (Drosophila)	ENSMUST00000115046	6.3 x (LII)	IHC	non-specific signal
	Cdh1	cadherin 1	ENSMUST00000000312	3.8 x (LIII)	IHC	non-specific signal
	Id1	inhibitor of DNA binding 1	ENSMUST00000109824	3.8 x (LII)	IHC	non-specific signal
**Layer II**	Slc13a3	thyrotropin releasing hormone receptor	ENSMUST00000110289	7.5 x (LI)	ISH	non-specific signal
	Neurod6	neurogenic differentiation 6	ENSMUST00000044767	5.5 x (LI)	IHC	non-specific signal
	Cart	CART prepropeptide	ENSMUST00000022150	3.7 x (LIII)	ISH	enriched in I and LIIa
	Npcd	neuronal pentraxin chromo domain	ENSMUST00000023060	3.6 x (LI)	ISH	enriched in LIIb and LIII
	Btbd3	BTB (POZ) domain containing 3	ENSMUST00000128656	3.4 x (LIII)	ISH	enriched in LII
	Prdm8	PR domain containing 8	ENSMUST00000112959	3.2 x (LI)	ISH	enriched in LI and LII
	Lmo3	LIM domain only 3	ENSMUST00000161450	3.0 x (LI)	ISH	enriched in LIIb and III
	Reelin	Reelin	ENSMUST00000162876	2.5 x (LIII)	IHC	enriched in LIIa
**Layer III**	Dpf1	D4, zinc and double PHD fingers family 1	ENSMUST00000128145	33 x (LI)	ISH	non-specific signal
	Sema3a	semaphorin 3A	ENSMUST00000095012	16 x (LI)	ISH	non-specific signal
	Neurod6	neurogenic differentiation 6	ENSMUST00000044767	15 x (LI)	IHC	non-specific signal
	SST	somatostatin	ENSMUST00000004480	12 x (LI)	IHC	expressed in subpopulation of cells in LIII
	Kcng1	potassium voltage-gated channel, subfamily G, member 1	ENSMUST00000109191	8.0 x (LI)	-	-
	PV	parvalbumin	ENSMUST00000005860	7.8 x (LI)	IHC	expressed in subpopulation of cells in LIII
	Barhl 1/2	BarH-like 2 (Drosophila)	ENSMUST00000086795	4.0 x (LII)	IHC	expressed in subpopulation of cells in LIII
	Lhx6	LIM homeobox protein 6	ENSMUST00000112960	4.0 x (LII)	ISH	enriched in LIII
	Foxp2	forkhead box P2	ENSMUST00000115475	3.3 x (LI)	IHC	expressed in subpopulation of cells in LIII
	Otx1/2	orthodenticle homolog 1 (Drosophila)	ENSMUST00000147486	2.1 x (LII)	IHC	expressed in subpopulation of cells in LIII

Supplementary Table 1. RNA deep sequencing identifies candidate genes enriched in piriform layers
Genes enriched in piriform layers I, II, and III were selected based on their fold-increase in expression levels compared to another layer (in parentheses), (p value < 0.05). Candidates were verified using Independent Component Analysis combined with Gene Set Enrichment Analysis. Gene expression data is available through the GEO repository (see Methods). ISH: RNA *in situ* hybridization. IHC: immunohistochemistry.
